# Antimicrobial activity of peptoids against Metallo-β-lactamase-producing *Klebsiella pneumoniae, Acinetobacter baumannii, Pseudomonas aeruginosa*, and other WHO priority pathogens, including *Candida auris*

**DOI:** 10.1093/jambio/lxaf031

**Published:** 2025-02-11

**Authors:** Shyam Kumar Mishra, Muhammad Yasir, Rajesh Kuppusamy, Edgar H H Wong, Alex Hui, Kristian Sørensen, Jennifer S Lin, Håvard Jenssen, Annelise E Barron, Mark Willcox

**Affiliations:** School of Optometry and Vision Science, Faculty of Medicine and Health, University of New South Wales, Sydney, NSW 2052, Australia; Department of Microbiology, Maharajgunj Medical Campus, Institute of Medicine, Tribhuvan University, Kathmandu 44600, Nepal; School of Optometry and Vision Science, Faculty of Medicine and Health, University of New South Wales, Sydney, NSW 2052, Australia; School of Optometry and Vision Science, Faculty of Medicine and Health, University of New South Wales, Sydney, NSW 2052, Australia; School of Chemistry, Faculty of Science, University of New South Wales, Sydney, NSW 2052, Australia; School of Chemical Engineering, Faculty of Engineering, University of New South Wales, Sydney, NSW 2052, Australia; School of Optometry and Vision Science, Faculty of Medicine and Health, University of New South Wales, Sydney, NSW 2052, Australia; Department of Bioengineering, School of Medicine and School of Engineering, Stanford University, Stanford, CA 94305, USA; Department of Bioengineering, School of Medicine and School of Engineering, Stanford University, Stanford, CA 94305, USA; Department of Science and Environment, Roskilde University, Roskilde 4000, Denmark; Department of Chemistry, University of Oslo, Oslo 0315, Norway; Department of Bioengineering, School of Medicine and School of Engineering, Stanford University, Stanford, CA 94305, USA; School of Optometry and Vision Science, Faculty of Medicine and Health, University of New South Wales, Sydney, NSW 2052, Australia

**Keywords:** biofilm, *Candida auris*, ESKAPEE pathogens, metallo-β-lactamase, multidrug resistance, peptidomimetic, synergy

## Abstract

**Aims:**

The World Health Organization has identified ESKAPE bacteria and *Candida auris* as priority pathogens, emphasizing an urgent need for novel antimicrobials to combat them. This study aimed to explore the therapeutic potential of antimicrobial peptidomimetics, specifically peptoids with sequence-specific *N*-substituted glycines, against ESKAPEE pathogens, including metallo-β-lactamase (MBL) producers, as well as *C. auris* strains.

**Methods and results:**

This study evaluated activity of the peptoids against the multidrug-resistant priority pathogens. The peptoid TM8 (with an *N*-decyl alkyl chain) demonstrated a geometric mean minimum inhibitory concentration (MIC) of 7.8 μg ml^−1^ against MBL-producing bacteria, and 5.5 μg ml^−1^ against *C. auris*. TM8 showed synergy with ciprofloxacin, enhancing its effectiveness 4-fold against NDM-1-producing *Klebsiella pneumoniae*. No antagonism was seen when TM8 was used with either conventional antibiotics or antifungals. Peptoids that had therapeutic indices below 3 were generally more hydrophobic, due to either alkyl chains or bromine. Scanning electron microscopy and live-dead staining assay on peptoid-treated *C. auris* confirmed morphological changes and killing activity, respectively. Furthermore, the peptoid could effectively inhibit biofilm formation by *C. auris*.

**Conclusion:**

Peptoids demonstrated antibacterial activity against ESKAPEE, particularly against MBL-producing Gram-negative bacteria. Additionally, they exhibited antifungal and anti-biofilm activities against *C. auris* strains.

Impact Statement Antimicrobial peptoids show promise as alternatives against multidrug-resistant ESKAPEE bacteria and the emerging yeast pathogen *Candida auris*. TM8 was active against vancomycin-resistant *Enterococcus faecalis*, methicillin-resistant *Staphylococcus aureus*, and carbapenemase-producing *Klebsiella pneumoniae, Acinetobacter baumannii, Pseudomonas aeruginosa*. TM8 exhibited anti-biofilm activity in *C. auris* and showed synergistic or additive interactions when used in combination with antimicrobials.

## Introduction

The World Health Organization (WHO) has published lists of antibiotic-resistant bacteria, including carbapenem-resistant *Enterobacteriaceae, Acinetobacter baumannii, Pseudomonas aeruginosa*, methicillin-resistant *Staphylococcus aureus*, vancomycin-resistant Enterococci, and the emerging fungal pathogen *Candida auris*, as priority pathogens (World Health Organization 2022, 2024). The ‘ESKAPE’ pathogens (*Enterococcus faecium, S. aureus, Klebsiella pneumoniae, A. baumannii, P. aeruginosa*, and *Enterobacter* species) alongside *C. auris*, are particularly significant due to their role in nosocomial infections and their diverse mechanisms of disease transmission, pathogenesis, and resistance (Rice 2008, Mishra et al. 2023, Politi et al. 2024). The ESKAPE pathogens list has been updated to include *Escherichia coli* (Ayobami et al. 2022), updating the moniker to be ‘ESKAPEE’. These pathogens are associated with infections of cardiovascular system, lower respiratory tract, gastrointestinal system, urinary tract, skin and soft tissue, bone and joints, as well as infections associated with indwelling medical devices (Pendleton et al. 2013, Miller and Arias 2024). ESKAPEE pathogens commonly exhibit resistance through mechanisms such as antimicrobial inactivation, target site modification, reduced penetration, reduced accumulation due to efflux pumps, and biofilm formation (De Oliveira et al. 2020). In 2019, the AMR-attributable deaths due to these pathogens have been estimated to be more than 330 000 (Miller and Arias 2024). On a global scale, antimicrobial resistance (AMR), in general, is projected to cause 10 million premature deaths annually.

A review on antibiotic resistance in nosocomial ESKAPEE infections in low- and lower middle-income countries has shown that pooled methicillin resistance in *S. aureus* was 48.4%, and pooled carbapenem resistance in *K. pneumoniae, P. aeruginosa, A. baumannii, Enterobacter* spp., and *E. coli* was 34.9%, 37.1%, 72.4%, 83.5%, and 16.6%, respectively (Ayobami et al. 2022). Although carbapenems can evade most β-lactamases, metallo-β-lactamases (MBLs), a class of carbapenemases, are capable of hydrolyzing a wide variety of other β-lactam antibiotics besides carbapenems (Queenan and Bush 2007). The dissemination of acquired MBLs, particularly *bla*_IMP_, *bla*_VIM_, and *bla*_NDM_, among Gram-negative bacteria has led to the emergence of extremely drug-resistant (XDR) phenotypes exhibiting cross-resistance to aminoglycosides, fluoroquinolones, and other antibiotic classes as well (Logan and Bonomo 2016, Boyd et al. 2020). These strains are resistant to β-lactamase inhibitors, including avibactam, relebactam, and vaborbactam, and their infections are associated with high 30-day mortality rates (Falcone et al. 2024). Though ceftazidime-avibactam and aztreonam, as well as aztreonam-avibactam, can show activity against *Enterobacterales* positive for extended-spectrum β-lactamase (ESBL), AmpC β-lactamase, Ambler class A carbapenemases (KPC, IMI), MBLs (NDM, VIM, and IMP), Ambler class D carbapenemases (OXA-48), they are not effective against difficult-to-treat *P. aeruginosa*, and carbapenem-resistant-*A. baumannii* (Macesic et al. 2025). Cefiderocol, a siderophore cephalosporin, demonstrates good antimicrobial activity against MDR Gram-negative bacteria. However, non-susceptibility has been observed in 24·9% (95% CI 16·6%–35·5%) of MBL-producing *Enterobacterales* and 40·9% (95% CI 34·5–55·4%) of NDM-producing *A. baumannii* isolates posing more problem (Karakonstantis et al. 2024).

Besides ESKAPEE bacterial pathogens, *C. auris* is a notorious nosocomial pathogen that has been reported from at least 62 countries. Key attributes, including challenges in accurate identification, high outbreak potential, high mortality rates ranging from 30% to 60% (Du et al. 2020), biofilm formation, resistance to standard antifungals such as azoles, and increasing resistance to echinocandins, underscore the urgent need for the development of novel antimicrobial approaches (Kim et al. 2024). In this context, antimicrobial peptides (AMPs) present a promising strategy to combat AMR in these pathogens targeting both planktonic cells as well as biofilms (Green and Bicker 2020, Salvado et al. 2024).

AMPs are integral components of the innate immune system across diverse organisms from animals and plants to fungi and bacteria (Chongsiriwatana et al. 2017). These peptides vary in number of amino acids (from 5 to >100) and are classified into various groups based on their amino acid composition, size and conformation. These AMPs are natural cationic peptides that carry a net positive charge (+2 to +13) and often contain up to 50% hydrophobic amino acids (Czyzewski et al. 2016). Their physiochemical properties, such as net charge, secondary structure, hydrophobicity, size, and amphipathicity determine their selectivity and potency (Hancock and Diamond 2000, Lee et al. 2018). However, they can be costly to produce, can trigger immune responses and cytotoxicity in the host, and are vulnerable to rapid degradation by proteases in the body (Andersson et al. 2016, Kalaiselvan et al. 2023). To address these limitations, non-natural peptidomimetics such as peptoids, peptide-peptoid hybrids, and β-peptoid–peptide hybrids have been developed (Chongsiriwatana et al. 2017).

Peptidomimetics are divided into four classes (A–D) based on their similarity to natural AMPs (Pelay-Gimeno et al. 2015). Class A mimetics closely resemble the original peptide with minimal local modifications in the parent structure (Lenci and Trabocchi 2020). The chimeric AMPs, melimine and Mel4, fall under class A peptidomimetics as they are derived from the natural peptides, melittin, and protamine. Class B peptidomimetics, on the other hand, are modified class A molecules with alterations in their backbone and side chains by non-natural amino acids or isolated small-molecule building blocks. These also encompass foldamers and peptoids that topologically align their side chains with the original peptide, but backbone is significantly modified (Lenci and Trabocchi 2020). Peptoids, class B antimicrobial peptidomimetics, are oligomers of *N*-substituted glycines, and this modification renders them resistant to proteolysis (Chongsiriwatana et al. 2017, Sara et al. 2024) offering significant clinical benefits. The peptoids mimic the amphipathic property of AMPs and, in some cases, can form three-sided polyproline type I helical secondary structures (Chongsiriwatana et al. 2008, Chongsiriwatana et al. 2011). AMPs and antimicrobial peptoids can operate via analogous mechanisms (Chongsiriwatana et al. 2017). Class C molecules are entities with extensively modified structures that fully substitute the peptide backbone and exhibit a small-molecule framework (Lenci and Trabocchi 2020). Class D peptidomimetics are different from others in that they resemble the bioactive peptide’s activity irrespective of their side chain functionalities (Lenci and Trabocchi 2020). Previous studies had focussed on the antimicrobial activity of peptoids against ocular pathogens such as *S. aureus* and *P. aeruginosa* (Sara et al. 2024) and bacteria from other infections, such as multidrug-resistant (MDR) *P. aeruginosa*, ESBL-producing *E.coli and K. pneumoniae*, and vancomycin-*resistant Enteroc. faecium and Enteroc. faecalis* (Czyzewski et al. 2016, Nielsen et al. 2022), but did not investigate whether the peptoids were active against MBL-producing Gram-negative bacteria. This is important as there is increase in the incidence of MBL in virulent Gram-negative pathogens, including *K. pneumoniae, A. baumannii*, and *P. aeruginosa*, making infections caused by such pathogens very difficult to treat (Mojica et al. 2022, Zakhour et al. 2024). Furthermore, the peptoid spectrum of action against yeasts has been limited to *C. albicans* and *C. neoformans* (Chongsiriwatana et al. 2011, Bicker and Cobb 2020) so far and their activity on *C. auris* has been unexplored. Therefore, this study aimed at exploring the activity of peptoids and AMPs against these and other ESKAPEE pathogens of various genetic profiles and resistance mechanisms, along with *C. auris* strains and their biofilms.

## Materials and methods

### Synthesis of peptoids and AMPs

The peptoids were synthesized through the standard submonomer solid-phase synthesis on Rink amide resin as previously described (Connolly et al. 2021, Nielsen et al. 2022, Sara et al. 2024). Acetylation and substitution were performed for 30 and 60 min, respectively, with overnight substitution using alkylamines. A cleavage mixture (95:2.5:2.5 trifluoroacetic acid, triisopropylsilane, and water) was applied for 30 min. Product purity was analysed via UPLC/MS using a Waters Symmetry300 C4 column with an aqueous acetonitrile gradient containing 0.1% trifluoroacetic acid. After purification, counterion exchange with 10 mmol l^−1^ HCl was conducted, and the compounds were lyophilized. The final purified products had >97% purity. Melimine and Mel4 (purity ≥95%) were synthesized following conventional solid-phase peptide synthesis protocols, and were procured from Auspep Peptide Company (Tullamarine, Victoria, Australia). The structure or sequence of the peptides and peptoids are presented in Table 1.

**Table 1.** Structure and properties of antimicrobial peptidomimetics used in the current investigations.

Class	Name (molecular mass)	Structure/Sequence
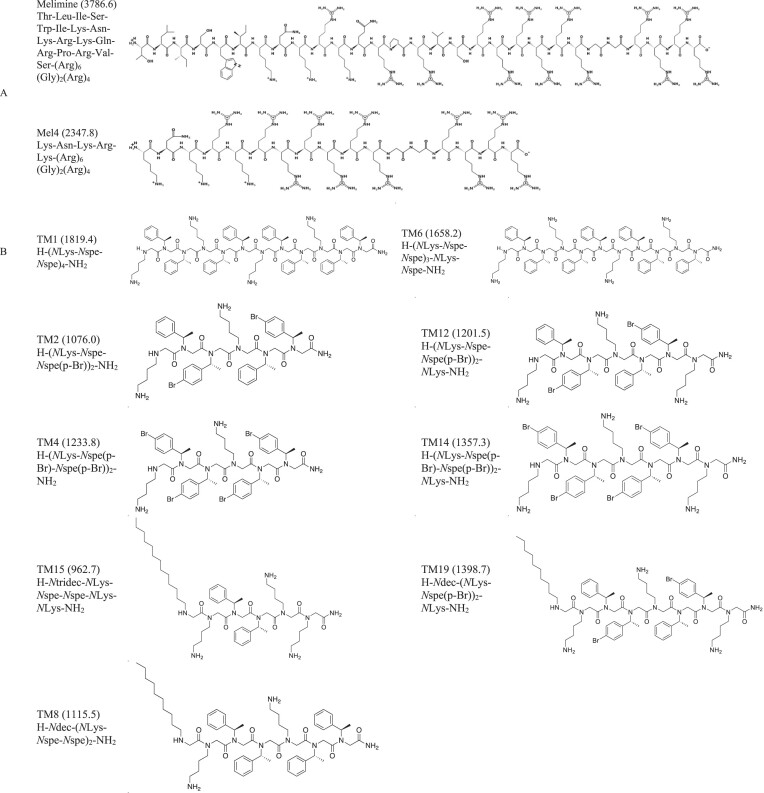		

### Antimicrobial sample preparation

Dry powders of peptoids were stored at −80°C before resuspending them in 1× phosphate buffered saline (PBS; NaCl- 8 g, Na_2_HPO_4_- 1.15 g, KCl- 0.2 g, KH_2_PO_4_- 0.2 g, in 1000 ml Milli-Q water; pH 7.2) to make stock solutions (2 mg ml^−1^) which were stored at −80°C. These stock solutions were later diluted in Mueller–Hinton Broth (MHB; Oxoid, Basingsoke, UK) for bacterial testing, and in Rose Park Memorial Institute (RPMI) 1640 with 3-(n-morpholino) propanesulfonic acid (MOPS) (Sigma–Aldrich, St. Louis, MO, USA) for *C. auris* to prepare working solutions at the desired concentrations. Antibiotics and antifungals (Sigma–Aldrich, USA) were dissolved in solvents recommended by Clinical and Laboratory Standards Institute (CLSI) (The Clinical and Laboratory Standards Institute 2008, Clinical and Laboratory Standards Institute 2024).

### Selection of microbes

The microbes in Table 2 were selected based upon their classification as priority pathogens by WHO and, where known, their antibiotic resistance profiles.

**Table 2.** List of microbes used in this study.

Bacteria	Strain	Possession of resistance gene(s)	Isolation source	Reference
*E nteroc. faecalis*	NCTC 13 379	*vanB*	Peritoneal fluid	(Tenover 1993)
*S. aureus*	04	NK	Catheter	(Thakur 1999)
*S. aureus*	38	NK	Cornea	(Bahatheg 2024)
*S. aureus*	105	*mecA*	Conjunctiva	(Afzal 2021)
*S. aureus*	117	NK	Cornea	(Afzal 2021
*K. pneumoniae*	JIE4212	*ampH, bla*_TEM-30_, *bla*_OXA-1_, *bla*_OXA-9_, *bla*_SHV-28_, *bla*_CTX-M-15_	-	(Fajardo-Lubián 2019)
*K. pneumoniae*	JIE2713	*aac(6′),bla*_CTX-M-15_ *, bla*_NDM-1_, *bla*_OXA-30_	-	(Shoma 2014, Venturini 2020 )
*K. pneumoniae*	JIE2709	*bla*_SHV_, *bla*_TEM_, *bla*_KPC_, *aac(6′)-Ib, aphA1, dfrA12, aadA2, sul1*	Urine	(Partridge 2015, Venturini 2020
*A. baumannii*	ATCC 19 606	*sul2, ant(3″)-IIa, bla*_ADC-25_, *bla*_OXA-98_	Urine	(Moffatt 2010, Zhu 2020)
*A. baumannii*	005	NK	Cornea	–
*A. baumannii*	006	NK	Cornea	–
*A. baumannii*	007	NK	Cornea	–
*A. baumannii*	008	NK	Cornea	–
*P. aeruginosa*	AR-1270	*aac(6′)-Ib-G, aadA1, aph(3″)-Ib, aph(3′)-IIb, bcr1, catB, dfrA5, ere*(A), *floR, floR2, fosA, bla*_GES-9_, *mexA, mexE, bla*_OXA-10_, *bla*_OXA-395_, *bla*_PDC-19A_, *qacEdelta1, rmtF2, sul1, tet*(G), *bla*_VIM-80_	Cornea	(Calvario and Shanks 2024)
*P . aeruginosa*	33	*aph(6)-Id, aph(3′')-Ib, crpP, blaPAO, bla*_OXA-50_, *qacEdelta1, sul1, tet(G), catB7, fosA*	Cornea	(Khan 2020b)
*P. aeruginosa*	216	*aph(3′)-IIb, crpP, bla*_OXA-50_ *, fosA, catB*	Cornea	(Khan 2020a)
*En. cloacae*	001	NK	Contact Lens	–
*E n. cloacae*	003	NK	Contact Lens	–
*En. cloacae*	005	NK	Conjunctiva	–
*E . coli*	005	NK	Contact lens	–
*E . coli*	0010	NK	Contact lens	–
*E . coli*	0011	NK	Contact lens	–
*E . coli*	0015	NK	–	–
**Fungi**				
*C. auris*	CBS10913	–	Auditory canal	(Satoh 2009, Sun 2024)
*C. auris*	CBS12373	(Fluconazole resistant)	Blood	(Kim 2009, Sun 2024

*vanB*, vancomycin; NK, not known; *mecA*, methicillin resistance gene; *bla*_TEM_, Temoniera; *bla*_OXA_, oxacillinase; *bla*_SHV_, sulfhydryl; *bla*_CTX-M_, cefotaximase-munich β-lactamase; *aac*, aminoglycoside acetyltransferase*; bla*_NDM_, New Delhi metallo-β-lactamase; *dfr*; dihydrofolate reductase; *bla*_KPC_, *Klebsiella pneumoniae* carbapenemases; *aph*, aminoglycoside phosphotransferase; *aad*, aminoglycoside adenyltransferase; *sul*, sulfonamide resistant dihydropteroate synthase*; ant*, aminoglycoside nucleotidyltransferase; *bla*_ADC_, Acinetobacter-derived cephalosporinase; *bcr*, bicyclomycin resistance; *catB*, chloramphenicol acetyltransferase B; *ere*(A), erythromycin esterase; *floR*, florfenicol resistance gene; *fosA*, fosfomycin resistance gene; *bla*_GES_, Guiana extended-spectrum β-lactamase; *mex*, multiple efflux; *bla*_PDC_, *Pseudomonas*-derived cephalosporinase; *qacEdelta*, Quarternary ammonium compound efflux gene delta; *rmt*, ribosomal methyltransferase; *tet*, tetracycline efflux pump; *bla*_VIM_, Verona integron-encoded metallo-β-lactamase; *crpP*, ciprofloxacin resistance protein; and *blaPAO, Pseudomonas aeruginosa* cephalosporinase.

### Antimicrobial activity of peptidomimetics and antibiotics

The antimicrobial activity was determined by broth microdilution assay as described previously (The Clinical and Laboratory Standards Institute 2008, Wiegand et al. 2008). Briefly, bacteria were suspended in cation-adjusted Mueller–Hinton broth (CAMHB; Oxoid, UK) for antibiotics, or MHB (Oxoid, UK) for peptidomimetics to obtain 5 × 10^5^ colony forming unit (CFU) ml^−1^. For *C. auris*, RPMI 1640 with MOPS (RPMI) was used to obtain the recommended 5 × 10^3^ CFU ml^−1^ for testing with conventional antifungals and peptidomimetics (The Clinical and Laboratory Standards Institute 2008). Antimicrobials were 2-fold diluted in CAMHB, MHB with 0.2% BSA and 0.01% acetic acid (for melimine/Mel4), MHB (for peptoids), or RPMI (for *C. auris*) in 96-well plates. Positive controls contained only microbes, and blanks contained only media. Microbial suspensions (100 µl) were added to wells, plates wrapped, and incubated at 37°C, 120 rpm for 16–20 h. After incubation, suspensions were removed, log10 diluted, and plated on Trypticase Soya Agar with phosphatidylcholine and Tween 80 to determine minimum bactericidal or fungicidal concentration (MBC or MFC). The minimum inhibitory concentration (MIC) of the antimicrobials for bacteria and fungi was determined as the lowest concentration of antibiotics or peptidomimetics that reduced viable cells by ≥90% (≥50% in case of *C. auris* for fluconazole), while the MBC or MFC was determined as the lowest concentration of the peptoid that reduced viable cell counts by 99.9%. The antibiograms of the bacterial pathogens were recorded following the interpretative chart provided by CLSI (Clinical and Laboratory Standards Institute 2024), while MIC susceptibility results for antifungals against *C. auris* were interpreted based on the US CDC guidelines (U.S. Centrers for Disease Control and Prevention 2024). A parallel control containing the final concentration of the solvent used to dissolve the antimicrobials (without the active compounds) confirmed the solvent’s non-toxicity, ensuring that the observed antimicrobial effects were solely attributable to the active compound. Geometric mean MIC of the peptoids were calculated to evaluate the broad overview of antimicrobial activity across the pathogens.

### Detection of metallo-β-lactamase by MBL strip

Meropenem-resistant Gram-negative bacterial isolates were subjected for MBL production using MIC Test Strip MBL strips (Liofilchem, TE, Italy), consisting of imipenem (IMI) and imipenem with EDTA (IMD). The test was performed as per the manufacturer’s instructions. Briefly, the strips were applied to a lawn culture of bacteria maintained at 1.5 × 10^8^ CFU ml^−1^ on Mueller–Hinton agar (Oxoid, UK) and incubated for 20 h at 37°C. If the MIC ratio of IMI and IMD was ≥8 or ≥3 log_2_ dilutions, the isolate was considered as MBL producer.

### Haemolysis assay

The haemolytic activity of the peptidomimetics was performed to determine mammalian cell safety. This assay used horse red blood cells (RBCs) (Sigma–Aldrich, USA) as described previously (Willcox et al. 2008, Chongsiriwatana et al. 2017, Yasir et al. 2019b). RBCs were washed three times with PBS at 470 *g* for 5 min. Peptoids (0.5–250 μg ml^−1^) were prepared in PBS and added to washed RBCs, then incubated at 37°C for 4 h. After incubation, cells were centrifuged at 1057 *g* for 10 min, and the supernatant’s OD at 540 nm was measured for lysis. PBS-only RBCs served as the negative control (no haemolysis), and Milli-Q water-treated RBCs as the positive control (100% lysis). Percent haemolysis was calculated as: (OD_T_ − OD_NC_)/(OD_PC_ − OD_NC_) × 100, where OD_T_, OD_NC_, and OD_PC_ are the absorbances of the test, negative, and positive controls, respectively. This experiment was performed in triplicate.

The therapeutic indices (TIs) of the compounds were calculated with the use of following formula:


\begin{eqnarray*}
&&{\mathrm{Therapeutic \, index}}\,\,( {\mathrm{TI}})\\
&&\quad = {\mathrm{H}}{{\mathrm{D}}_{50}}/{\mathrm{geometric \, mean \, MIC \, where \, H}}\\
&&\quad{{\mathrm{D}}_{50}}\,\,{\mathrm{is \, the}}\,\,50\%
\,\,{\mathrm{haemolysis \, dose}}.
\end{eqnarray*}

The overall TI was calculated as the ratio of the HD_50_ to the geometric mean of the MIC against the microbes tested (Chongsiriwatana et al. 2008, Bacalum and Radu 2015, Li et al. 2023). Similarly, selectivity ratio (SR) of the antimicrobials was calculated as the ratio of the HD_10_ (10% haemolysis dose) and the geometric mean MIC (Chongsiriwatana et al. 2008, Andreev et al. 2018).

### Antimicrobial synergy

The checkerboard microdilution test was performed to determine the fractional inhibitory concentration (FIC) of selected antimicrobials and assess whether the combination had synergistic, antagonistic, or indifferent effects (Anon 2016) against strains of *K. pneumoniae* or *C. auris*. Different antibiotics with high MIC were tested to evaluate potential synergy with TM8. Additionally, a strain susceptible to ciprofloxacin was tested to observe the outcome of its combination with TM8 as a representative case from susceptible isolates. Briefly, TM8 and different antibiotics or fluconazole were serially diluted to form a gradient of combination concentrations in 96-well microtitre plates. Then, 10 µl of bacteria (5 × 10^6^ CFU ml^−1^) or *C. auris* (2.5 × 10^4^ CFU ml^−1^) were added to each well, except for the sterility control well. Wells in the first column contained only the antibiotic (or fluconazole), while the first row received only TM8 at different concentrations. The sterility control wells contained only MHB (or RPMI in case of *C. auris*), and the growth control contained 110 µl of microbial inoculum with no antimicrobials. Subsequently, the plates were incubated for 18- 24 hours at 36 ± 1 °C.

The FIC for each combination interaction was calculated as follows:


\begin{eqnarray*}
&&\mathrm{FIC \, of \, agent \, A}\,\,( \mathrm{FI} \mathrm{C}_{\mathrm{A}} )\\
&&\quad= \mathrm{MIC \, of \, agent \, A \, in \, combination}/ \mathrm{MIC \, of \, agent \, A \, alone}\\
&& \mathrm{FIC \, of \, agent \, B}\,\,( \mathrm{FI} \mathrm{C}_{\mathrm{B}} )\\
&&\quad= \mathrm{MIC \, of \, agent \, B \, in \, combination}/ \mathrm{MIC \, of \, agent \, B \, alone}.
\end{eqnarray*}

Then, the summation of FIC (ƩFIC) was calculated for each antimicrobial combination as follows: ƩFIC = FIC_A_ + FIC_B_. The results were interpretated as follows: Synergy if ƩFIC ≤ 0.5; Additive if 0.5<ƩFIC ≤ 1; Indifference if 1>ƩFIC ≤ 4; and Antagonism if ƩFIC > 4 (Odds 2003). All antimicrobial synergy experiments were repeated in at least two independent runs.

### Effect of TM8 on *C. auris* cells

The effect of TM8 on the membrane integrity of yeast cells was evaluated by flow cytometry using the fluorescent dyes SYTO 9 and PI, with modifications to a previously described method (Yasir et al. 2019a). Briefly, the MIC of TM8 against *C. auris* CBS10913 (at a cell density of 10^6^ CFU ml^−1^) was determined to be 15.6 µg ml^−1^. At this cell density, yeast cells in PBS (pH 7.2) were exposed to TM8 for 90 min at 37 °C. Afterwards, the cells were stained with SYTO 9 (7.5 µmol l^−1^) and PI (30 µmol l^−1^) for an additional 15 min in the dark at room temperature. Following washing, samples were then analysed for membrane integrity on a BD FACSymphony^TM^ A3, with a minimum of 10 000 events recorded per sample.

Further, a suspension of *C. auris* CBS 10913 was prepared as described for the MIC test and processed for scanning electron microscopy (SEM) as previously described (Oliveira et al. 2019) with some modifications. The isolate was treated with TM8 at its MIC for 90 min, with each treatment performed in triplicate. The aliquots were then pooled into 1.5 ml Eppendorf tubes. The *C. auris* pellet was fixed in 2.5% glutaraldehyde in 0.1 mol l^−1^ sodium cacodylate buffer for 1 h at room temperature. A 500 µl volume of the fixed *C. auris* suspension was allowed to settle on a 13 mm glass coverslip for 10 min. The sample was then washed three times with 0.1 mol l^−1^ sodium cacodylate buffer, followed by additional washes with deionized water, and finally dehydration by graded ethanol series (30%, 50%, 70%, 90%, and two changes of 100%). The *C. auris* sample on the coverslip was subjected to critical point drying (CPD) in a Leica EM CPD300. For SEM imaging, a platinum coating was applied to the sample surface using Q300T D Plus. The samples were then imaged with a NanoSEM 230 at the Mark Wainwright Analytical Centre, UNSW Sydney.

*Candida auris* CBS 10913 (∼2 × 10^6^ CFU ml^−1^) was prepared, and the MIC of TM8 was determined. Killing kinetics (time-kill) were evaluated as described previously (Lum et al. 2015). Yeast cells (1–2 × 10^6^ CFU ml^−1^) in RPMI were treated with TM8 at 1 × and 4 × MIC and incubated at 35°C for 24 h with agitation. At set intervals (0, 0.5, 1, 1.5, 2, 2.5, 3, 4, 5, 6, 12, and 24 h), 100 µl samples were serially diluted in PBS and plated on Sabouraud dextrose agar (SDA; Oxoid, UK) with 0.5% polysorbate 80 (Chem-supply, SA, Australia). CFUs were enumerated, with a yeast growth control processed similarly. Results were presented from three independent runs.

### Effect of TM8 on biofilm formation of *C. auris*

Previous studies have tested the antibiofilm activity of some peptoids against bacterial biofilms (Lin et al. 2022, Nielsen et al. 2022). In this study, we specifically evaluated the antibiofilm activity of TM8 against *C. auris* biofilm. Among the two *C. auris* strains, *C. auris* CBS12373 was selected for biofilm inhibition testing because it was previously identified as a strong biofilm producer (Larkin et al. 2017), and its resistance to fluconazole. Biofilm formation was assessed following previously established methods (Chandra et al. 2008).

To measure the ability of TM8 to reduce the initial adhesion and subsequent biofilm formation of *C. auris* CBS12373, 5 × 10^4^ yeast cells in RPMI were added to 96-well flat bottom microtiter plates with TM8 (62.5–0.12 µg ml^−1^) or plain RPMI (negative control) or fluconazole (positive control; 1024–2 µg ml^−1^) and allowed to adhere for 2 h at 37°C. Following a single wash in PBS to remove planktonic cells, fresh medium was added, and the plates incubated at 37°C for 48 h, followed by washing two times with PBS. Finally, 3-(4,5-Dimethylthiazol 2-yl)-2,5-diphenyltetrazolium bromide (MTT) reduction assay was performed to observe the metabolic activities of *C. auris*.

To determine the ability of TM8 to reduce initial biofilm formation, 2 × 10^5^ cells of *C. auris* in RPMI were allowed to adhere to wells of 96-well flat bottom microtiter plates for 4 h at 37°C, followed by a PBS wash and addition of TM8 (125–0.06 µg ml^−1^) or RPMI alone, with a further 48-h incubation. To assess disruption of mature biofilms, 2 × 10^5^ cells were cultured for 2 h in 96-well flat bottom microtiter plates at 37°C, washed, then incubated for 48 h in fresh media to form mature biofilm. After double-washing, TM8 (125 to 0.06 µg ml^−1^) or RPMI alone was added for 24 h at 37°C. For each activity test, the metabolic activities of the yeast cells in biofilms were measured by MTT reduction assay after washing the disrupted biofilm with PBS (Srivastava and Ahmad 2020).

Another set of experiments was conducted under similar conditions similar to those used for MTT assay. Briefly, after the biofilms in plates were treated with TM8 for the required duration and washed with PBS, the crystal violet assay was performed, as described previously (Macia et al. 2014), by adding 0.1% crystal violet to wells to measure the biofilm biomass to determine initial biofilm inhibitory and mature biofilm eradication concentrations.

### Sorbitol protection assay

The MIC of TM8 against *C. auris* CBS 10913 was evaluated in RPMI with and without sorbitol (0.8 mol l^−1^; Sigma, St. Louis, MO, USA) under the same conditions as described above to evaluate any increase in MIC till 7 days. Caspofungin served as a positive control for the assay (Leite et al. 2014).

### Ergosterol binding assay

The MIC of TM8 against *C. auris* CBS 10913 was determined in the presence and absence of ergosterol (200 µg ml^−1^; Sigma–Aldrich, St. Louis, MO, USA) in RPMI under the same conditions as described above to assess any increase in MIC within 48 h. Amphotericin B was tested in parallel as a positive control (Ramesh et al. 2023).

### Statistical analysis

Experiments were performed in duplicate with three independent biological replicates. The quantitative data collected from independent experiments are expressed as the mean ± standard deviation (SD) or standard errors of the means. Statistical analysis was performed using independent *t*-test, or one way ANOVA for group comparisons, with Kruskal–Wallis test applied for non-parametric data. Statistical significance was observed for *P*-values <0.05. The figures were plotted with GraphPad Prism version 10.0.3.

## Results

### Antibiotic resistance profile of the bacterial pathogens

First, the MICs of the bacterial isolates were determined. Table 3 summarizes the antibiotic sensitivity profiles across various antibiotics. The results showed that most of the bacteria were resistant to a broad array of antibiotic classes, establishing them as MDR or XDR. An ocular isolate of methicillin-resistant *S. aur eus* (MRSA), SA105, was resistant to all standard antibiotics, except vancomycin, which remains a last resort treatment. However, an *Enteroc. faecalis* isolate was vancomycin-resistant, with an MIC of 32 µg ml^−1^. Among Gram-negative bacteria, three isolates of *K. pneumoniae* were resistant to extended-spectrum cephalosporin, monobactams, and ciprofloxacin, but remained susceptible to polymyxins. Similarly, all *A. baumannii* isolates were resistant to meropenem. The test panel also included an XDR *P. aeruginosa* isolate, AR-1270, associated with keratitis linked to contaminated artificial tears, which led to blindness and death in several cases in the USA (Grossman et al. 2024, Sundermann et al. 2024). This isolate carried both Verona integron-encoded MBL and Guiana ESBL genes, highlighting its high-level resistance (Calvario and Shanks 2024).

**Table 3.** MIC of antibiotics against ESKAPEE pathogens.

	MIC of antibiotics (µg ml^−1^)								
Gram-positive strains	Penicillin	Macro-lide	Tetra-cycline		Fluoro-quinolone	Phenicol	Cepha-mycin		Glyco-peptide
	Pen	Ery	Min	Cip		Chl	Cefox		Van
*Enteroc. faecalis* NCTC 13 379	4	4	0.5		0.125	**64**	NR		**32**
*S. aureus* 04	**16**	0.5	0.25		0.25	2	0.5		1
*S. aureus* 38	**16**	1	0.5		0.25	4	0.5		1
*S. aureus* 105	**32**	**32**	**16**		**128**	**32**	**16**		1
*S. aureus* 117	**16**	1	0.5		0.5	4	0.5		4
	MIC of antibiotics (µg ml^−1^)								
Gram-negative strains	Fluoro-quinolone	Amino-glycoside	Extended-spectrum cephalosporin		Mono-bactam	Carba-penem	Poly-myxin		Pheni-col
	Cip	Gen	Cefot	Cefaz	Azt	Mer	PmB	Col	Chl
*K. pneumoniae* JIE4212	**64**	**128**	**>512**	**>512**	**>128**	1	1	1	**32**
*K. pneumoniae* JIE2713	**128**	**>128**	**>512**	**>512**	**128**	**>64**	1	1	**32**
*K. pneumoniae* JIE2709	**128**	0.5	**32**	**32**	**>128**	**32**	1	1	**>32**
*A. baumannii* ATCC 19 606	1	4	8	8	NR	0.5	2	2	NR
*A. baumannii* 005	**64**	>**128**	**512**	**512**	NR	**>64**	0.25	0.25	NR
*A. baumannii* 006	**>128**	**>128**	**512**	**512**	NR	**64**	0.25	0.25	NR
*A. baumannii* 007	**>128**	**>128**	**512**	**512**	NR	**64**	1	1	NR
*A. baumannii* 008	**128**	**>128**	**512**	**512**	NR	**64**	1	1	NR
*P. aeruginosa* AR-1270	**>128**	>**128**	NR	**>512**	**>128**	>**64**	2	1	NR
*P. aeruginosa* 33	**128**	>**128**	NR	**32**	**128**	**4**	2	1	NR
*P. aeruginosa* 216	0.5	NR	NR	2	4	1	**16**	**16**	NR
*En. cloacae* 001	1	4	4	4	4	1	2	2	**32**
*En. cloacae* 003	0.06	0.5	8	4	4	1	1	1	**32**
*En. cloacae* 005	0.06	0.25	**128**	**128**	**16**	0.25	2	2	**32**
*E. coli* 005	0.06	0.25	0.25	0.25	0.5	2	0.25	0.25	8
*E. coli* 0010	0.06	0.25	2	2	2	2	2	2	8
*E. coli* 0011	0.06	0.5	4	4	4	1	2	2	1
*E. coli* 0015	0.06	0.5	0.5	0.5	0.5	2	0.25	0.25	2

Numbers in bold indicate resistance to those antibiotics. NR, not recommended by CLSI (2024), and so not tested; Pen, penicillin; Ery, erythromycin; Min, minocycline; Cip, ciprofloxacin; Chl, chloramphenicol; Cefox, cefotaxime; Van, vancomycin; Gen, gentamicin; Cefot, cefotaxime; Cefaz, ceftazidime; Azt, aztreonam; Mer, meropenem; PmB, polymyxin B; Col, colistin sulfate.

### Evaluation of peptidomimetics against ESKAPEE pathogens

Table 4 presents the mean MIC of peptidomimetics against the ESKAPEE pathogens. The MICs for the class B peptidomimetic TM peptoids were generally lower than those for class A peptidomimetics (Table 4). Among the peptoids, TM8 showed the lowest MIC values against the ESKAPEE pathogens. Its MIC ranged from 3.9 to 7.8 µg ml^−1^ against Gram-positive bacteria (*Enteroc. faecalis* and *S. aureus*), while it ranged from 3.9 to 15.6 µg ml^−1^ against Gram-negative bacteria, with a geometric mean MIC of 7.3 µg ml^−1^ across all bacteria. For the XDR *P. aeruginosa* AR-1270 strain, both TM8 and TM4 exhibited the lowest MICs, with values of 15.6 µg ml^−1^ each.

**Table 4.** MIC of antimicrobial peptidomimetics against ESKAPEE pathogens.

Strains	MIC of peptidomimetics µg ml^−1^ (µmol l^−1^)										
	Melimine	Mel4	TM1	TM2	TM4	TM6	TM8	TM12	TM14	TM15	TM19
*Enteroc. faecalis* NCTC 13 379	500 (122)	500 (213)	31.3 (17.2)	31.3 (29.0)	7.8 (6.3)	31.25 (18.8)	7.8 (7.0)	7.8 (6.5)	7.8 (5.7)	31.3 (32.4)	31.3 (22.3)
*S. aureus* 004	31.3 (8.2)	62.5 (26.6)	7.8 (7.2)	3.9 (3.2)	7.8 (4.7)	7.8 (4.7)	3.9 (3.5)	3.9 (3.3)	7.8 (5.7)	7.8 (8.1)	7.8 (5.6)
*S. aureus* 38	31.3 (8.2)	62.5 (26.6)	7.8 (7.2)	7.8 (7.2)	3.9 (3.2)	7.8 (4.7)	3.9 (3.5)	7.8 (6.5)	3.9 (2.9)	7.8 (8.1)	3.9 (2.8)
*S. aureus* 105	31.3 (8.2)	62.5 (26.6)	3.9 (2.1)	7.8 (7.2)	3.9 (3.2)	7.8 (4.7)	3.9 (3.5)	7.8 (6.5)	3.9 (2.9)	7.8 (8.1)	3.9 (2.8)
*S. aureus* 117	31.3 (8.2)	62.5 (26.6)	7.8 (7.2)	7.8 (7.2)	3.9 (3.2)	7.8 (4.7)	3.9 (3.5)	3.9 (3.3)	7.8 (5.7)	7.8 (8.1)	7.8 (5.6)
*K. pneumoniae* JIE4212	250 (66.0)	250 (106.5)	15.6 (8.6)	62.5 (58.1)	7.8 (6.3)	15.6 (9.4)	7.8 (7.0)	125 (103.8)	31.3 (23.0)	62.5 (64.9)	31.3 (22.3)
*K. pneumoniae* JIE2713	125 (33.0)	250 (106.5)	62.5 (34.4)	125 (116.2)	31.3 (25.3)	62.5 (37.7)	15.6 (14.0)	125 (103.8)	7.8 (5.7)	125 (129.8)	62.5 (44.7)
*K. pneumoniae* JIE2709	500 (122)	62.5 (26.6)	15.6 (8.6)	125 (116.2)	31.3 (25.3)	31.3 (18.8)	15.6 (14.0)	125 (103.8)	62.5 (45.9)	125 (129.8)	31.3 (22.3)
*A. baumannii* ATCC 19 606	62.5 (16.5)	125 (53.2)	3.9 (2.1)	31.3 (29.0)	15.6 (12.6)	7.8 (4.7)	3.9 (3.5)	125 (103.8)	31.3 (23.0)	62.5 (64.9)	7.8 (5.6)
*A. baumannii* 005	125 (33.0)	250 (106.5)	3.9 (2.1)	7.8 (7.2)	15.6 (12.6)	7.8 (4.7)	7.8 (7.0)	62.5 (51.8)	15.6 (11.4)	62.5 (64.9)	7.8 (5.6)
*A. baumannii* 006	62.5 (16.5)	62.5 (26.6)	3.9 (2.1)	7.8 (7.2)	15.6 (12.6)	7.8 (4.7)	3.9 (3.5)	125 (103.8)	15.6 (11.4)	62.5 (64.9)	7.8 (5.6)
*A. baumannii* 007	125 (33.0)	250 (106.5)	3.9 (2.1)	31.3 (29.0)	15.6 (12.6)	7.8 (4.7)	3.9 (3.5)	62.5 (51.8)	31.3 (23.0)	62.5 (64.9)	7.8 (5.6)
*A. baumannii* 008	250 (66.0)	250 (106.5)	7.8 (7.2)	15.6 (8.6)	7.8 (6.3)	15.6 (9.4)	7.8 (7.0)	62.5 (51.8)	31.3 (23.0)	31.3 (32.5)	7.8 (5.6)
*P. aeruginosa* AR-1270	500 (122)	500 (213)	31.3 (17.2)	31.3 (29.0)	15.6 (12.6)	31.3 (18.8)	15.6 (14.0)	125 (103.8)	62.5 (45.9)	31.3 (32.5)	31.3 (22.3)
*P. aeruginosa* 33	62.5 (16.5)	62.5 (26.6)	15.6 (8.6)	31.3 (29.0)	15.6 (12.6)	31.3 (18.8)	3.9 (3.5)	3.9 (3.3)	15.6 (11.5)	15.6 (16.2)	7.8 (5.6)
*P. aeruginosa* 216	500 (122)	250 (106.5)	15.6 (8.6)	15.6 (14.5)	7.8 (6.3)	15.6 (9.4)	7.8 (7.0)	31.3 (25.9)	15.6 (11.5)	15.6 (16.2)	7.8 (5.6)
*En. cloacae* 001	125 (33.0)	250 (106.5)	15.6 (8.6)	31.3 (29.0)	15.6 (12.6)	15.6 (9.4)	7.8 (7.0)	15.6 (13.0)	15.6 (11.5)	15.6 (16.2)	15.6 (11.2)
*En. cloacae* 003	125 (33.0)	250 (106.5)	62.5 (34.4)	31.3 (29.0)	7.8 (6.3)	15.6 (9.4)	7.8 (7.0)	3.9 (3.3)	62.5 (45.9)	62.5 (64.9)	15.6 (11.2)
*En. cloacae* 005	250 (66.0)	250 (106.5)	15.6 (8.6)	15.6 (8.6)	15.6 (12.6)	15.6 (9.4)	15.6 (14.0)	15.6 (13.0)	62.5 (45.9)	31.3 (32.5)	31.3 (22.3)
*E. coli* 005	250 (66.0)	250 (106.5)	15.6 (8.6)	125 (116.2)	15.6 (12.6)	62.5 (37.7)	7.8 (7.0)	125 (103.8)	31.3 (23.0)	62.5 (64.9)	7.8 (5.6)
*E. coli* 0010	250 (66.0)	250 (106.5)	15.6 (8.6)	62.5 (58.1)	15.6 (12.6)	62.5 (37.7)	15.6 (14.0)	125 (103.8)	31.3 (23.0)	62.5 (64.9)	7.8 (5.6)
*E. coli* 0011	125 (33.0)	62.5 (26.6)	31.3 (17.2)	62.5 (58.1)	7.8 (6.3)	15.6 (9.4)	15.6 (14.0)	31.3 (25.9)	31.3 (23.0)	62.5 (64.9)	15.6 (11.2)
*E. coli* 0015	125 (33.0)	62.5 (26.6)	31.3 (17.2)	62.5 (58.1)	15.6 (12.6)	62.5 (37.7)	7.8 (7.0)	62.5 (51.8)	31.3 (23.0)	15.6 (16.2)	31.3 (22.3)
Geometric mean MIC	132.8 (35.1)	149.8 (63.8)	13.0 (7.2)	26.1 (24.2)	11.2 (9.1)	17.6 (10.6)	7.3 (6.6)	31.2 (26.0)	19.9 (14.6)	32.2 (33.4)	12.6 (9.0)

### Determination of MBL-production in Gram-negative bacteria

The meropenem-resistant isolates, *K. pneumoniae* JIE2713, *K. pneumoniae* JIE2709, *A. baumannii* 005, *A. baumannii* 006, *A. baumannii* 007, *A. baumannii* 008, *P. aeruginosa* AR-1270, and *P. aeruginosa* 33 were tested for MBL production. All *A. baumannii* isolates, along with *K. pneumoniae* JIE2713 and *P. aeruginosa* AR-1270, were found to produce MBL indicated by an MIC reduction of ≥8-fold for imipenem in the presence of EDTA (Table 5). While the genetic determinants of MBL-production in the *A. baumannii* isolates are not known, the MBL-producing *K. pneumoniae* JIE2713 possesses the *bla*_NDM_ and *P. aeruginosa* AR-1270 carries *bla*_VIM_.

**Table 5.** MBL detection in carbapenem-resistant Gram-negative bacteria.

Strain	Imipenem IMI (μg ml^−1^)	Imipenem + EDTA IMD (μg ml^−1^)	MBL Interpretation
*K. pneumoniae* JIE2713	128	1	**Positive**
*K. pneumoniae* JIE2709	16	16	Negative
*A. baumannii* 005	64	4	**Positive**
*A. baumannii* 006	64	8	**Positive**
*A. baumannii* 007	96	8	**Positive**
*A. baumannii* 008	48	2	**Positive**
*P. aeruginosa* AR-1270	16	2	**Positive**
*P. aeruginosa* 33	8	1.5	Negative

### Synergy between antibiotics and peptoid for *K. pneumoniae*

The combined interaction of ciprofloxacin with TM8 demonstrated FIC of 0.023 for *K. pneumoniae* JIE2713 with ƩFIC of 0.273 (Table 7). It further showed additive activity with meropenem, cefotaxime, aztreonam, and gentamicin.

### Haemolysis assay and therapeutic index

**Figure 1.**
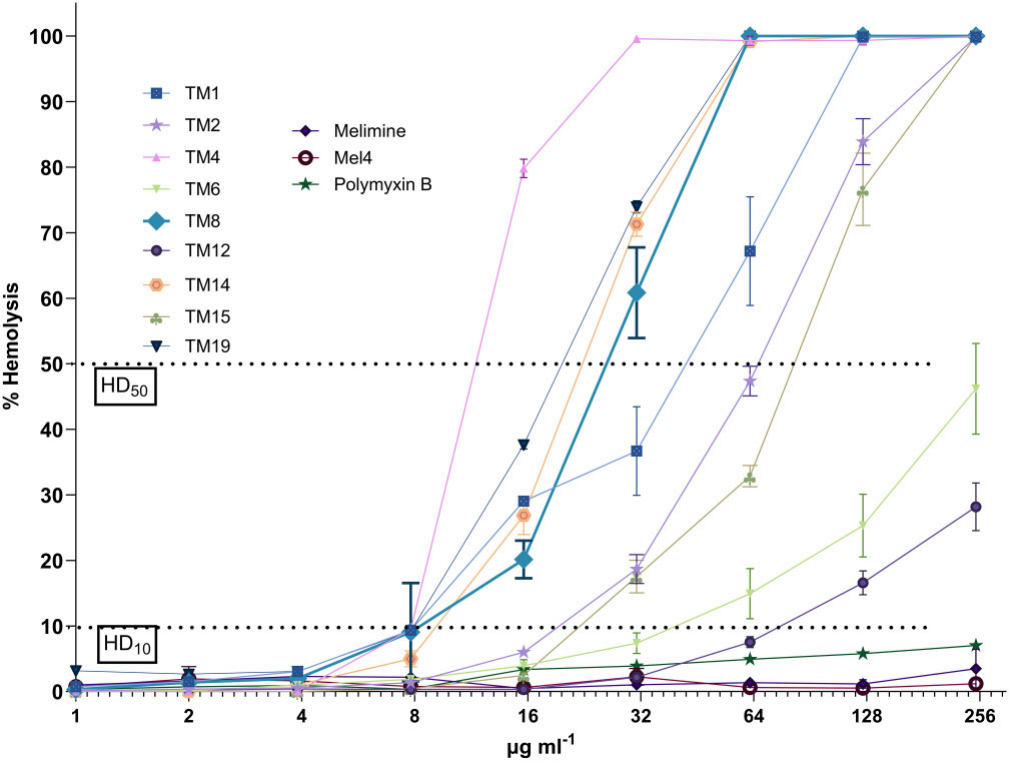
Haemolytic activities of peptide mimics and antibiotic showing HD50 and HD10. Data represent mean ± standard error of mean, *n* = 3 independent experiments.

Fig. 1 illustrates the haemolytic activities of peptidomimetics and polymyxin B with the HD_50_ and HD_10_ cut off points shown. This information was utilized in Table 6, along with best fit curve models, to calculate the TIs and SRs of the peptidomimetics for the tested bacterial strains. In this assay assessing the TIs of peptoids across all bacteria, the order from greatest to least was TM6 and TM12, TM8, TM1, TM15, TM2, TM19, TM14, and TM4 (Table 6). For the SRs, the order was TM12, TM6, TM8, TM2, TM4 and TM15, TM1 and TM19 and TM14 (Table 6). The class A peptidomimetics melimine and Mel4 had high HD_10_ and HD_50_ of >256 µg ml^−1^ and so exact TIs or SRs could not always be calculated (Table 6). With respect to the MBL-producers, TM6 followed by TM12, TM1, and TM8 had the therapeutic index >3, whereas only TM6, TM12, and TM8 had SR ≥1. For the *Enterobacteriaceae*, the highest therapeutic index was given for TM6 > TM12 > TM8, with the remaining TMs having TIs below 2.0. Only TM12, TM6, and TM8 showed selectivity index >1 for the *Enterobacteriaceae*.

**Table 6.** TIs and SRs of peptidomimetics against bacteria.

			Geometric mean in µg ml^−1^			Therapeutic index			Selectivity ratio		
Peptide mimics	HD_10_, µg ml^−1^ (µmol l^−1^)	HD50, µg ml−1 (µmol l−1)	MIC_E_	MIC_MBL_	MIC _All bacteria_	TI_E_	TI _MBL_	TI _All bacteria_	SR_E_	SR _MBL_	SR _All bacteria_
Melimine	>256 (>67.6)	>256 (>67.7)	189.5	162.1	132.8	>1.4	>1.6	>1.9	>1.4	>1.6	>1.9
Mel4	>256 (>109)	>256 (>109)	164.9	162.1	149.8	>1.6	>1.6	>1.7	>1.6	>1.6	>1.7
TM1	8 (4.4)	44.8 (24.6)	23.7	11.0	13.0	1.9	4.1	3.4	0.3	0.7	0.6
TM2	20 (18.6)	64 (59.5)	58.3	28.7	26.0	1.1	2.2	2.5	0.3	0.7	0.8
TM4	8 (6.5)	12.4 (10.1)	14.6	17.0	11.2	0.9	0.7	1.1	0.5	0.5	0.7
TM6	43.2 (26.1)	>256 (>154.4)	29.1	18.6	17.6	>8.8	>13.8	>14.5	1.5	2.3	2.5
TM8	8 (7.2)	27.2 (24.4)	11.0	7.8	7.3	2.5	3.5	3.7	0.7	1.0	1.1
TM12	80 (66.4)	>256 (212.6)	47.4	62.5	31.2	>5.4	>4.1	>8.2	1.7	1.3	2.6
TM14	9.6 (7.0)	24 (17.6)	31.3	24.1	19.9	0.8	1.0	1.2	0.3	0.4	0.5
TM15	23.6 (24.5)	86.4 (89.7)	50.8	52.6	32.2	1.7	1.6	2.7	0.5	0.4	0.7
TM19	8 (5.7)	20.8 (14.8)	20.6	14.3	12.6	1.0	1.5	1.6	0.4	0.6	0.6

HD_10_ and HD_50_, 10% and 50% haemolytic doses, respectively; MIC_E_, MIC of *Enterobacteriaceae* (*E. coli, K. pneumoniae*, and *E. aerogenes*); MIC_MBL_, MIC of MBL-producing bacteria; TI_E_, therapeutic index with respect to *Enterobacteriaceae* bacteria (*E. coli, K. pneumoniae*, and *E. aerogenes*); TI_MBL_, therapeutic index with respect to MBL-producing bacteria; selectivity ratio (SR) = HD_10_/MIC or geometric mean MIC; SR_E_, selectivity ratio of *Enterobacteriaceae* bacteria (*E. coli, K. pneumoniae*, and *E. aerogenes*); SR_MBL_, selectivity ratio of MBL-producing bacteria.

### Evaluation of peptidomimetics against *C. auris*

In the current study, TM4 had the lowest MIC for both strains of *C. auris*, with TM15 having the highest MIC with strain CBS10913 only. TM19 and TM8 also had relatively low MICs for both strains. Table 8 gives the MIC of conventional antifungals, class A peptidomimetics and peptoids against two strains of *C. auris*. There are currently no established breakpoints for traditional antifungals against *C. auris*, although tentative break-points have been published (https://www.cdc.gov/fungal/candida-auris/c-auris-antifungal.html) for each of the three traditional antifungals. Therefore, based on CDC criteria, strain CBS 12373 is resistant to fluconazole, but susceptible to amphotericin B and caspofungin, and strain CBS 10913 is susceptible to all three traditional antifungals. Interestingly, strain CBS 12373 had lower MICs for melimine, Mel4 and peptoids compared to strain CBS 10913.

For both *C. auris* strains, the combination of TM8 and fluconazole resulted in an additive effect; however, the modulation factor of fluconazole by 0.5× MIC of TM8 was 16, which resulted in the MIC of fluconazole resistant strain CBS12373 (MIC = 8 µg ml^−1^ in presence of TM8) falling below the breakpoint for fluconazole of 32 µg ml^−1^ as shown in Table 7.

**Table 7.** Checkerboard assay of TM8 with different antibiotics against *K. pneumoniae*, and antifungal against *C. auris*.

Isolates	Drug B (Antibiotic)	MIC of TM8 (µg ml^−1^)	MIC of TM8 + DrugB (µg ml^−1^)	FIC_TM8_	MIC of DrugB (µg ml^−1^)	MIC of DrugB + TM8 (µg ml^−1^)	FIC_DrugB_	ƩFIC	Outcome	MF* of TM8	MF of antibiotic
*K. pneumoniae* JIE2709	Meropenem	15.625	7.8125	0.5	32	4	0.125	0.625	Additive	2	8
	Cefotaxime	15.625	7.8125	0.5	32	16	0.5	1	Additive	2	2
	Ciprofloxacin	15.625	7.8125	0.5	128	64	0.5	1	Additive	2	2
*K. pneumoniae* JIE2713	**Ciprofloxacin**	**15.625**	**3.90 625**	**0.25**	**128**	**32**	**0.023**	**0.273**	**Synergy**	**4**	**4**
	Cefotaxime	15.625	7.8125	0.5	1024	512	0.5	1	Additive	2	2
	Aztreonam	15.625	7.8125	0.5	128	64	0.5	1	Additive	2	2
	Gentamicin	15.625	7.8125	0.5	1024	512	0.5	1	Additive	2	2
*C. auris* CBS10913	Fluconazole	7.813	3.9063	0.5	1	0.0625	0.0625	0.563	Additive	2	16
*C. auris* CBS12373	Fluconazole	3.9	1.95	0.5	128	8	0.0625	0.563	Additive	2	16

* MF, modulation factor = MIC of single antimicrobial/MIC of antimicrobials in combination.

Table 9 demonstrates the therapeutic index and SRs of the peptidomimetics against the two *C. auris* strains. Both melimine and Mel4 had similar relatively high therapeutic index and SR (>11.6). Similar to the results with the ESKAPEE bacteria (Table 8 ), TM6 had the highest therapeutic index against *C. auris*. This was followed by TM15 and TM12, while all other TMs had TIs of ≤ 7.5. Similarly, the SR was highest for TM15, closely followed by TM6 and TM12, with the remaining TMs showing values ≤ 4.

**Table 8.** MIC of conventional antifungals and peptidomimetics against *C. auris*.

*C. auris* strains	MIC µg ml^−1^ (µmol l^−1^)														
	Conventional antifungals			Peptidomimetics											
	Azole	Polyene	Echinocandin	Class A (AMPs)			Class B (Peptoids)								
	Fluconazole	Amphotericin B	Caspofungin	Melimine	Mel4		TM1	TM2	TM4	TM6	TM8	TM12	TM14	TM15	TM19
CBS 10 913	1 (3.3)	0.25 (0.27)	0.125 (114.4)	125 (33.0)	125 (53.2)	_	15.6 (8.6)	31.25 (29)	2 (1.6)	31.25 (18.8)	7.8 (7.0)	62.5 (51.9)	15.6 (11.5)	250 (259.6)	3.9 (2.8)
CBS 12 373	**128 (417.9)**	0.25 (0.27)	0.25 (228.7)	3.9 (1.03)	3.9 (1.7)	_	3.9 (2.1)	3.9 (3.6)	2 (1.6)	2 (1.2)	3.9 (3.5)	3.9 (3.2)	3.9 (2.9)	3.9 (4.0)	2 (1.4)

**Bold**, indicates resistance based on break-points at https://www.cdc.gov/fungal/candida-auris/c-auris-antifungal.html.

**Table 9.** TIs and SRs of peptidomimetics against *C. auris*.

Peptide mimics	HD_10_ in µg ml^−1^ (µmol l^−1^)	HD_50_ in µg ml^−1^ (µmol l^−1^)	Geometric mean in µg ml^−1^	Therapeutic index	Selectivity ratio
			MIC_CA_	TI_CA_	SR_CA_
Melimine	>256 (>67.6)	>256 (>67.7)	22.1	>11.6	>11.6
Mel4	>256 (>109)	>256 (>109)	22.1	>11.6	>11.6
TM1	8 (4.4)	44.8 (24.6)	7.8	5.7	1.4
TM2	20 (18.6)	64 (59.5)	11.1	5.8	1.8
TM4	8 (6.5)	12.4 (10.1)	2	6.2	4
TM6	43.2 (26.1)	>256 (>154.4)	7.9	>32.4	5.5
TM8	8 (7.2)	27.2 (24.4)	5.5	4.9	1.5
TM12	80 (66.4)	>256 (212.6)	15.6	>16.4	5.1
TM14	9.6 (7.0)	24 (17.6)	7.8	3.1	1.2
TM15	23.6 (24.5)	86.4 (89.7)	3.9	22.2	6
TM19	8 (5.7)	20.8 (14.8)	2.8	7.5	2.9

TI_CA_, therapeutic index with respect to *Candida auris* strains; SR_CA_, selectivity ratio of *C. auris* strains.

### SEM, flow cytometry, and time-kill assay of peptoid treated *C. auris*

**Figure 2.**
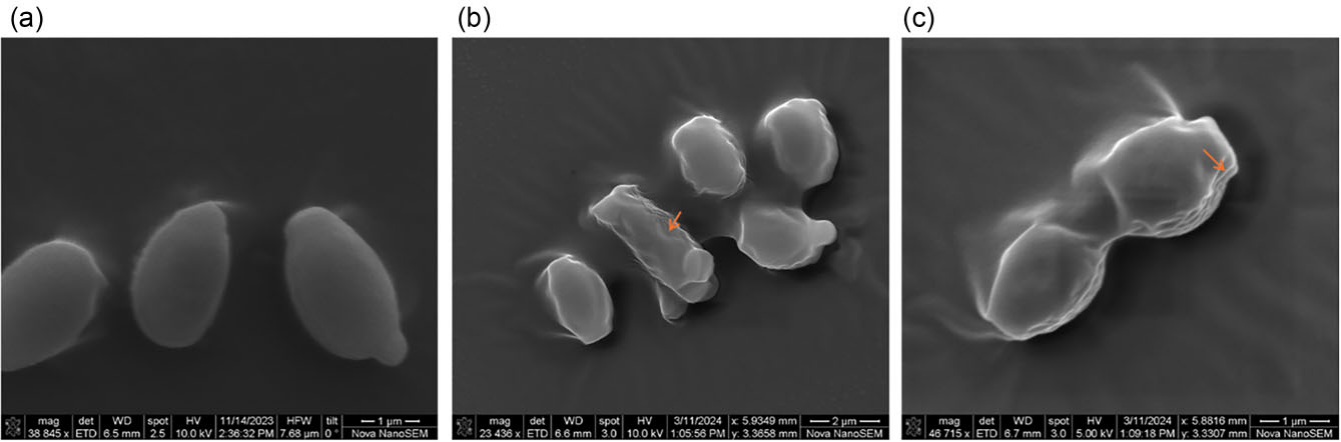
SEM images of *C. auris* CBS 10913 cells: (a) untreated control cells at ×39 000 magnification; (b, c) cells treated with TM8 at their MIC for 90 min, at ×23 000 and ×49 000 magnifications, respectively. The treated cells exhibit irregular morphology and rough surfaces indicated by orange-coloured arrows in contrast in contrast to the smooth appearance of the control yeast cells. Observations were made on three independent repeats.

**Figure 3.**
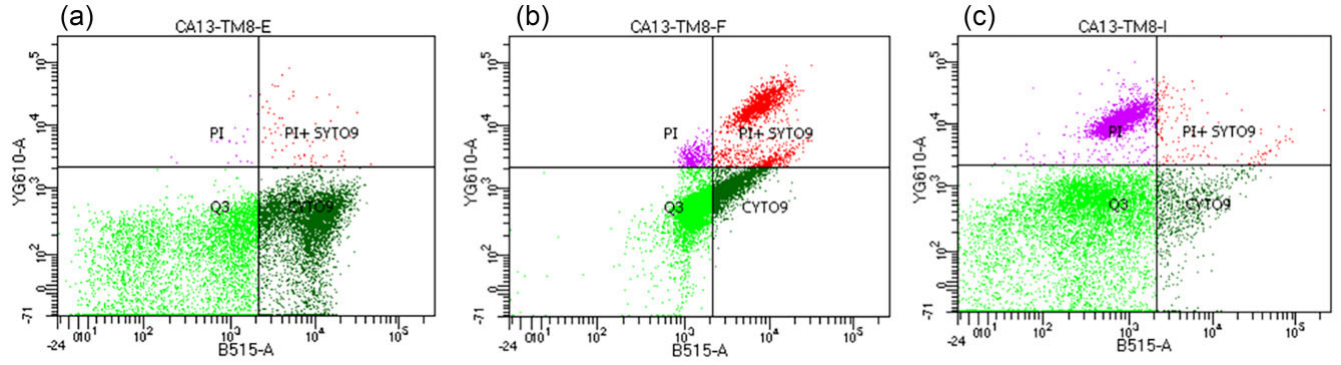
Membrane permeabilization of *C. auris* CBS 10913 determined by flow cytometry with SYTO 9 and propidium iodide staining on treatment with (a) 1× PBS (negative control), (b) TM8 at its MIC, and (c) 1% Triton X-100 (positive control) (Fig. 3).

SEM demonstrated that TM8 treatment resulted in *C. auris* having an irregular cell surface (Fig. 2). To support the cell lytic activity of the peptoid, *C. auris* was simultaneously exposed to SYTO 9 and PI in the presence of TM8 and a control of 1% Triton X-100. PI functions as a membrane-impermeable dye that fluoresces only upon entering the cells and binding to nucleic acids, whereas SYTO 9 traverses cell membranes irrespective of their integrity. In the presence of TM8, the SYTO 9 fluorescent signal of *C. auris* decreased from 51.7% (Fig. 3a) to 25.5% (Fig. 3b). The number of dead cells was 3.9% compared to 0.2% in the cells treated only with PBS (negative control). There was also a remarkable increase in population of cells doubly stained with SYTO 9 and PI, from 0.7% to 21.5% in the presence of TM8 (Fig. 3a and b). This indicates that the cells were injured after exposure to the peptoid. In cells treated with 1% Triton X-100 (positive control), 27.3% of cells were dead.

**Figure 4.**
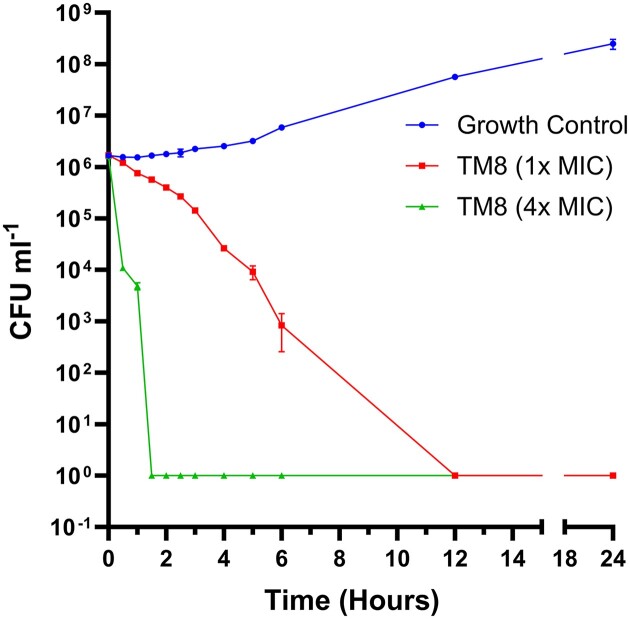
The time-kill kinetics of TM8 against *C. auris* CBS 10913. Samples taken at 30-min interval and CFUs were determined on SDA medium supplemented with 0.5% polysorbate 80. The values represent the means of the independent triplicate runs.

The time-kill assay on *C. auris* CBS 10913 showed that TM8, at 1× MIC, achieved a 3-log reduction within 6 h and completely killed the yeast cells within 12 h. At 4× MIC, TM8 exhibited fungicidal activity within 2 h *(P*-values <0.05) (Fig. 4).

### Antibiofilm activity of TM8 on *C. auris*

To determine the biofilm inhibitory concentrations of TM8 and fluconazole, the antimicrobial concentrations that reduced the *C. auris* CBS 12373 yeast cells by ≥90% were determined. TM8 demonstrated a lower “Biofilm Tolerance Factor-Inhibition” concentration, calculated as the ratio of minimum biofilm inhibitory concentration (MBIC) to the MIC of the antimicrobial required to inhibit growth of 90% of cells (Table 10). The MICs of TM8 and fluconazole for the *C. auris* suspension used in the antibiofilm study were 15.6 and 512 µg ml^−1^, respectively. TM8 exhibited anti-adherence, initial biofilm inhibition, and mature biofilm eradication activities at 8-fold, 2-fold, and 4-fold higher concentrations, respectively, compared to its MIC when assessed using the MTT assay. The mature biofilm inhibition data has been provided in supplementary file S1. Initial biofilm inhibition and mature biofilm eradication were further evaluated using the crystal violet staining method, which determined the MBIC for initial biofilm as 31.25 µg ml^−1^ and the MBEC for mature biofilm as 125 µg ml^−1^ (data not shown in table).

**Table 10.** Biofilm inhibitory concentrations of TM8 and fluconazole against *C. auris*.

Strain	Anti-microbial (µg ml^−1^)	MIC_90_ (µg ml^−1^)	Anti-adherence (BPC) (µg ml^−1^)	Initial biofilm MBIC (µg ml^−1^)	Mature biofilm MBEC (µg ml^−1^)	Biofilm tolerance factor-prevention (BPC/MIC)	Biofilm tolerance factor-inhibition (MBIC/MIC)	Biofilm tolerance factor-eradication (MBEC/MIC)
*C. auris* CBS12373	TM8	15.6	125	31.25	62.5	8	2	4
	Fluconazole	512	4096	2048	2048	8	4	4

### Ergosterol binding and sorbitol protection assays

To assess the binding ability of TM8 to either the cytoplasmic membrane or the cell wall, ergosterol binding and sorbitol protection assays, respectively, were performed on *C. auris* CBS 10913. In the ergosterol binding assay, the MIC of TM8 increased from 7.8 to 31.25 µg ml^−1^ in the presence of exogenous ergosterol, suggesting that TM8 targets the fungal cell membrane. A similar increase in MIC was observed with the positive control, amphotericin B, which also acts on the fungal membrane, showing a five-fold increase from 0.25 µg ml^−1^. In the sorbitol protection assay, the MIC of TM8 remains unchanged at 7.8 µg ml^−1^, indicating that it does not act on the fungal cell wall. In contrast, the positive control, caspofungin, showed an increase in MIC from 0.25 to 1 µg ml^−1^ in the presence of sorbitol.

## Discussion

The surge in AMR is occurring at an accelerated rate, which highlights the increasingly urgent need for the development of novel antimicrobial agents. In this study, the antimicrobial activity of two different classes of peptidomimetics were evaluated against several WHO priority pathogens, with a focus on their actions against MBL-positive *A. baumannii, K. pneumoniae, P. aeruginosa*, and a yeast, *C. auris*, which had not been previously investigated. As, overall, the MICs of peptoids were much lower than that for the class A peptidomimetics (melimine and Mel4) against both ESKAPEE pathogens and *C. auris* strains, this study focussed on the protease-resistant class B peptidomimetics (peptoids) for their antimicrobial, antibiofilm, and synergistic properties with conventional antimicrobials.

Out of the nine peptoids, TM1 is the most studied previously which consists of a mixture of two monomers, α-chiral aromatic (*S*)-*N*-(1-phenylethyl)glycine (*N*spe) and cationic lysine-like *N*-(aminobutyl)glycine (*N*Lys) residues (Patch and Barron 2003). The antimicrobial activity of TM1 has been attributed to its cationicity, and a helical and facially amphipathic structure (Bicker and Cobb 2020). TM8, TM15, and TM19 featured terminal alkyl tails of either *N*-decyl or *N*-tridecyl, while all of them contained chiral aromatic *N*spe along with *N*Lys residues. Similarly, TM2, TM4, TM12, TM14, and TM19 were halogenated (brominated) peptoids. Among the peptoids, TM12 and TM15 had the highest geometric mean MICs against all bacteria tested including *Enterobacteriaceae* while TM8 had the lowest geometric mean MIC overall. The MIC of TM8 ranged from 3.9 to 15.6 µg ml^−1^ for all the pathogens tested. This confirms a previous study showing that the MIC of TM8 against MDR ESKAPE pathogens was <13 µg ml^−1^ (Nielsen et al. 2022). In the case of bacteria, when the structure of TM8 was altered to TM15 by shortening (*N*Lys-*N*spe-*N*spe)_2_ to *N*Lys-*N*spe-*N*spe and adding *N*Lys*-N*Lys and changing the 10-carbon alkyl chain to a 13-carbon chain, there was a dramatic increase in the geometric mean MIC by 4-fold showing decrease in antimicrobial activity. While the *N*tridec tail in TM15 may enhance interaction with bacterial membranes at low concentrations (Moule et al. 2024), and the addition of *N*Lys increases its cationicity, the decrease in hydrophobicity due to the reduced number of *N*spe residues likely contributes to its lower antimicrobial activity as compared to TM8. Similarly, altering TM8 to TM19 by adding bromine to two of the *N*spe moieties and adding an extra *N*Lys increased the geometric mean MIC by nearly 2-fold. However, removing the 10-carbon alkyl chain from TM8 while adding bromine to each *N*spe to produce TM4 only increased the geometric mean MIC by 1.5-fold. The reduced activity of TM4 as compared to TM8, despite the presence of bromine, likely resulted from the absence of the alkyl chain. This alteration also increased cytotoxicity, conferring a therapeutic index of 1.1 against bacteria. When TM4 was changed to TM12 by the addition of *N*Lys and removal of two bromines from *N*spe, there was again a dramatic increase in geometric mean of 3-fold. TM4 which is tetra-brominated had a lower geometric mean MIC as compared to TM2 which is di-brominated. A similar trend was observed with TM14 (tetra-brominated) and TM12 (di-brominated) against ESKAPEE pathogens. Previous studies have also reported that bromination enhances antibacterial activity due to increase in hydrophobicity. However, this activity is influenced by the sequence and length of the peptoid (Molchanova et al. 2020), Similarly, cytotoxicity also increases with the increase in halogen atom as evidenced by reduced therapeutic index in TM4 and TM14. Nevertheless, the antimicrobial activity of peptoids declines after a certain level of hydrophobicity (Molchanova et al. 2020). The MIC values for the *P. aeruginosa* and *S. aureus* strains in the current study are very similar to those reported in a previous report (Sara et al. 2024). In a previous study, TM8 demonstrated similar or improved activity compared to TM1 against ESKAPE pathogens, including *Enterococcus* sp., *A. baumannii*, and *P. aeruginosa* (Nielsen et al. 2022). In the current study, TM8 had equal or greater activity when compared to TM1.

Lipopeptoids can self-assemble into micellar macromolecules due to the hydrophobic tail domains driven by the intermolecular hydrophobic interactions. TM8 has been found to self-assemble in to an ellipsoidal form at concentrations as low as 1 μg ml^−1^ (Nielsen et al. 2022), well below its MICs, allowing TM8 to potentially function as a vehicle-free self-controlled delivery system (Yang et al. 2014). Peptoids that undergo self-assembly in such forms exhibit pronounced activity against ESKAPE pathogens, whereas those forming worm-like micelles demonstrate reduced antimicrobial effects. In a previous study, TM9 and TM10, brominated analogues of TM8 with the same or increased number of fatty acid tails, formed some worm-like micelles in addition to ellipsoidal micelles, and exhibited reduced activity compared to TM8. This further supports the idea that self-assembly in the ellipsoidal form is crucial for their activity. Notably, the peptoids self-assembled at concentrations below their MICs against all microbes. The same study also showed that if the peptoid is short and does not form ellipsoidal micelles, it does not possess antibacterial activity (Nielsen et al. 2022). When compared to TM8 and TM19, the lipopeptoid TM15, which also possesses a fatty acid tail that increases the affinity for anionic bacterial membranes, demonstrated weaker antimicrobial activity as evidenced by a higher geometric mean MIC against all microbes, suggesting that the compound’s length may also influence its activity. However, further studies are recommended to explore how ellipsoidal structure affects antimicrobial activity.

The activity of TM compounds against MBL producing strains has not been previously reported. New-Delhi-Metallo-β-lactamases (NDMs) are a type of MBL that have at least 60 different variants. The variant NDM-1 shows the highest host spectrum. They can hydrolyze all β-lactam antibiotics except aztreonam unless they possess ESBL. One of the tested bacterial strains, *K. pneumoniae* JIE2713, possesses both ESBL and MBL (NDM-1), along with another carbapenemase (Oxacillinase, OXA-30) thus showing resistance to carbapenems, cephalosporins, and monobactams (aztreonam). This strain is also resistant to ciprofloxacin and gentamicin. Although taniborbactam, a novel β-lactamase inhibitor, has shown promising results against MBL-producing strains over the last 5 years (Mehta Pooja et al. 2016), recently an MBL-producing strain resistant to taniborbactam has been reported (Le Terrier et al. 2023). Another MBL-inhibitor, xeruborbactam, was found to be less effective against MBL-producing *P. aeruginosa* due to MexAB-OprM efflux pump (Le Terrier et al. 2024). Therefore, any novel molecule which can counteract the effects of such strains is of great clinical value. In the current study, TM8 and TM14 were the most potent peptoids against *K. pneumoniae* JIE2713, as were the polymyxins. TM4 and TM8 had the lowest MICs against *bla*_VIM-80_ producing *P. aeruginosa* AR-1270, while TM1, TM8, and TM19 exhibited MICs ≤7.8 µg ml^−1^ against MBL phenotypes of *A. baumannii*. A single MBL-producing *K. pneumoniae* JIE2709 was tested for synergy of TM8 and meropenem, with an additive result, indicating that TM8 could act in concert with meropenem against this bacterium.

Given TM8’s strong antimicrobial activity, we further explored its interaction with different antibiotics against *bla*_KPC_ and *bla*_NDM_ possessing MDR *K. pneumoniae*. No antagonism was seen in any isolate with any combination of TM8 with other antibiotics. While the phenomenon behind the synergy between the TM8 and ciprofloxacin in *K. pneumoniae* JIE2713 was not examined, we hypothesize that TM8’s potential membrane compromising action at sub-MIC levels may facilitate ciprofloxacin’s entry into bacterial cells. Once inside, ciprofloxacin can exert its effect by inhibiting DNA gyrase, thereby halting DNA replication, or causing fragmentation of bacterial DNA (Silva et al. 2011). Additionally, peptoids have been found to penetrate bacterial membranes, bind to DNA and form flocculates leading to cell death (Chongsiriwatana et al. 2017), which could also potentiate ciprofloxacin’s activity. It is hypothesized that self-assembled structures of these peptoids disassociate upon contact with anionic bacterial membranes, resulting in fast bacterial membrane permeabilization (Nielsen et al. 2022).

Having evaluated the effect of the peptoids on ESKAPEE bacteria, their effects on two different isolates of *C. auris* were evaluated. TM4, TM19, and TM8 had MICs <10 µg ml^−1^ against both *C. auris* strains. This may indicate that the hydrophobicity of TMs is important in activity against *C. auris*, as TM4 was brominated, and changing TM4 to TM19 removed 2 bromines from *N*spe but added an alkyl chain, and then TM19 to TM8 removed all bromines and shortened the alkyl chain. The two strains of *C. auris* had dissimilar MICs across the TM compounds, with strain CBS 10913 often having a much higher MIC to the compounds. These differences may be due to differences in the membrane lipidomes of the isolates, especially as the TMs were shown to affect the membrane integrity of *C. auris* by electron microscopy and cell staining (Figs 2 and 3). Previous studies have shown that the membrane lipidome of *C. auris* CBS10913T was distinct to that from an isolate resistant to antifungals such as fluconazole and ketoconazole, with changes in phosphatidylcholine, phosphatidylglycerol, phosphatidylserine, phosphatidylinositol, and phosphatidylethanolamine, amongst others (Shahi et al. 2020). Another study found that fluconazole resistant strains of *C. auris* had higher concentrations of sphingolipids compared to amphotericin resistant strains (Kumar et al. 2021). While these differences may not be directly related to differences in resistance to TM compounds, they do indicate an avenue for future research to understand the susceptibility differences between *C. auris* strains.

As TM8 showed promising activity against both bacteria and *C. auris*, further studies were conducted to investigate its mode of action on yeast cells by performing ergosterol binding and sorbitol protection assays. Exogenous ergosterol prevented the binding of TM8 to ergosterol in the yeast membranes, thereby increasing its MIC, but sorbitol had no effect. Since sorbitol acts as an osmoprotectant stabilizing fungal protoplasts, this result suggests that TM8 targets the *C. auris* membrane and probably does not act on the yeast’s cell wall (Leite et al. 2014).

Another way microbes can acquire tolerance and become resistant to antimicrobials is by the production of biofilms. In biofilms, microbes switch from planktonic state to sessile form, where they are encased in an extracellular polymeric substance matrix, thereby being protected from stressful environmental conditions (Di Fermo et al. 2024). Most ESKAPEE pathogens and *C. auris* form antimicrobial tolerant biofilms associated with device-associated infections (Kean and Ramage 2019, Sarkar et al. 2024). The antibiofilm activity of some of these peptoids has already been studied against ESKAPE pathogens, with TM8 showing significant inhibition of biofilm formation in *S. aureus, K. pneumoniae, P. aeruginosa*, and *En. cloacae* at 1.56 μg ml^−1^ (Nielsen et al. 2022). However, the antibiofilm activity of peptoids against *C. auris* has not been explored. Therefore, we investigated the antibiofilm effect of TM8 on a reference strain of *C. auris*. TM8 had better efficacy in disrupting premature biofilms compared to fluconazole. *Candida auris* biofilm consists of a mannan-glucan complex, which causes sequestration of antimicrobials, preventing it from entering the cells (Dominguez et al. 2019). Furthermore, genes encoding for efflux pumps have been noted in *C. auris* biofilms mediating resistance to antifungals. However, our peptoid was effective in preventing biofilm formation as well as in disrupting preformed biofilms. TM8 required only 2–8-fold higher concentration, than those effective against planktonic cells, to prevent or eradicate *C. auris* biofilm. In contrast, another study reported 4- to 64-fold greater concentrations of AMPs than against planktonic cells to exhibit antibiofilm activity (Hashemi et al. 2018). Interestingly, certain AMPs show broad-spectrum antibiofilm activity at concentrations below the MIC for planktonic cells (de la Fuente-Núñez et al. 2015). Further mechanistic studies are needed to elucidate the antibiofilm activity of our peptoid; although, a study on *C. albicans* has shown that different peptoids can inhibit hyphae formation with changes in the morphology of the nucleus and nucleolus, with inclusion of lipidic bodies into the nucleus as determined by soft X-ray tomography (Uchida et al. 2009).

## Conclusions

This study identified TM8 as the most effective peptoid investigated, having activity against all the extensively drug-resistant isolates, including MBL-producing bacteria and *C. auris*. TM8 demonstrated superior antimicrobial activity, and a better therapeutic index compared to the well-studied peptoid TM1. Additionally, TM8 synergized effectively with ciprofloxacin, while no antagonism was observed when tested in combination with either antibiotics or antifungals. This research marks the first time these peptoids have been studied against *C. auris*, with TM8 showing good activity against both the planktonic cells and biofilms of *C. auris*. Overall, these findings position TM8 as a potent antimicrobial agent. Further experiments focussing on its activity against different subclasses of MBLs and clades of *C. auris* should be conducted, as well as its *in vivo* efficacy and safety assessed.

## Limitations

Only a single isolate of *Enteroc. faecalis* was included in this study, although a previous study has shown similar activities with other strains (Czyzewski et al. 2016). The different number of strains from each genus may limit the interpretations of geometric mean MIC values obtained. Similarly, the activity of peptidomimetics on different clades of *C. auris* was not studied. In addition, although the only polymyxin B resistant strain, *P. aeruginosa* 216, tested had a relatively low overall MIC to the TMs (average 14.7 µg ml^−1^), there is a potential for cross-resistance between polymyxins and the peptoids due to their possibly similar modes of action, and so testing additional strains is needed. Cytotoxicity studies were conducted solely using horse RBCs. Therefore, testing on different cell lines and RBCs from other species would provide an insight into the toxicity of these compounds, and help to generalize the findings. Furthermore, *in vivo* infection models should also be utilized in future research to better evaluate the therapeutic potential of these peptoids.

## Supplementary Material

lxaf031_Supplemental_File

## Data Availability

Supplementary file S1: Biofilm disruption.
